# Supplementary material for: Longitudinal assessment of neuronal 3D genomes in mouse prefrontal cortex
Source: Nat Commun. 2016 Sep 6;7:12743. doi: 10.1038/ncomms12743 (PMC5025847; doi:10.1038/ncomms12743)
Supplement: Supplementary Information — Supplementary Figures 1-6, Supplementary Tables 1-2 [file ncomms12743-s1.pdf]

SUPPLEMENTARY FIGURE 1

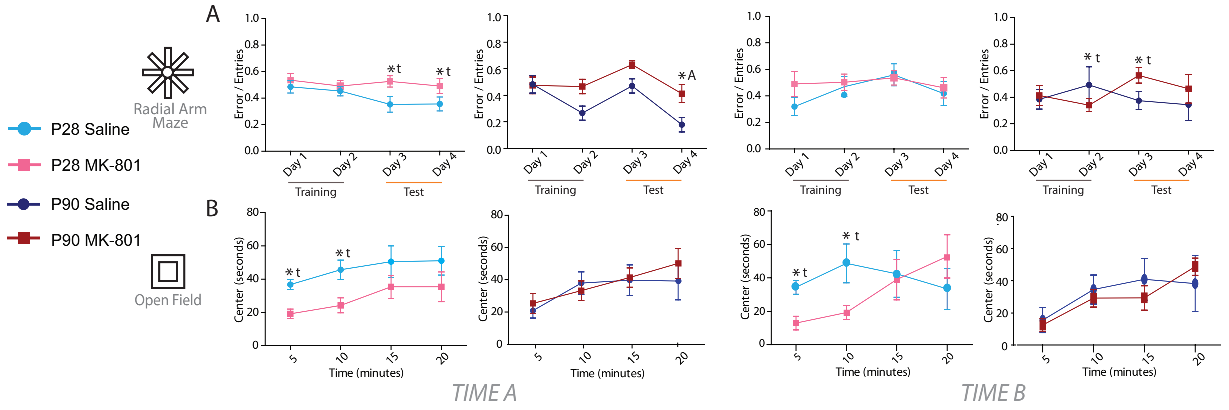

Supplementary Figure 1: **Behavioral alterations after repeated MK-801 exposure** (**A**) Radial arm maze and (**B**) open field test in P28 and P90 treated mice, as indicated. For TIME A, N=10-11 animals/age group and treatment. For Time B, N=5-7 animals/age group and treatment. Data in (A,B) shown as mean±S.E.M. Significant effects by drug for (**A**) TIME A/P28 mice, 2-way ANOVA (treatment x day), drug $F_{(1,3)}$=8.252, P=0.0097; one-tailed t-test day 3 P=0.013, day 4 P=0.049. TIME A/P90 mice, 2-way ANOVA (treatment x day), drug $F_{(1,3)}$=10.66, P=0.0062, post-hoc Bonferroni saline:MK801 day 4 P<0.05, t=2.916. TIME B/P90 mice, one tailed t-test, day 2 P=0.023, day 3=0.035; for (**B**) TIME A/P28 mice, 2-way ANOVA (drug x time interval), drug F=6.752, P=0.018, df =1, one tailed t-test P=0.003 for time 5min and P=0.004 for time 10 min. TIME B/P28, one tailed t-test P=0.001 time 5 min and P=0.002 time 10 min.

Supplementary Figure 2

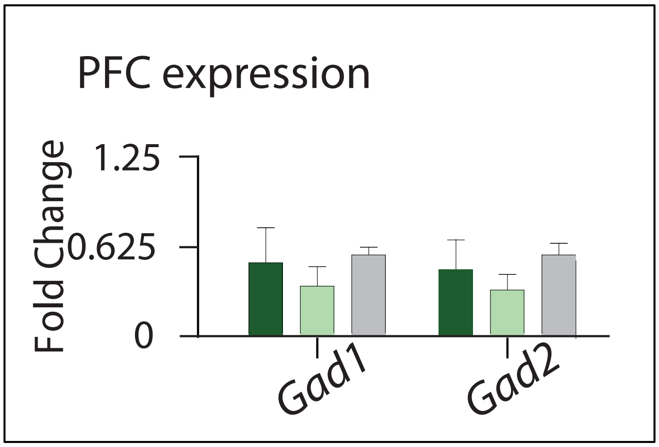

Supplementary Figure 2: Minimal changes in *Gad1* and *Gad2* expression in HSV TALE^{Gad1}Dam injected cortex (rescaled y-axis/same data as shown in Figure 1D): qRT-PCR to quantify *Gad1* and *Gad2* RNA expression 2, 7 and 10 days after injection of HSV amplicon encoding TALE^{Gad1}Dam. Dam RNA was assayed with two independent primer pairs Dam.1 and Dam.2 Data expressed as fold-change and shown after normalization to 18S rRNA. Data shown as mean±S.E.M.

Supplementary Figure 3

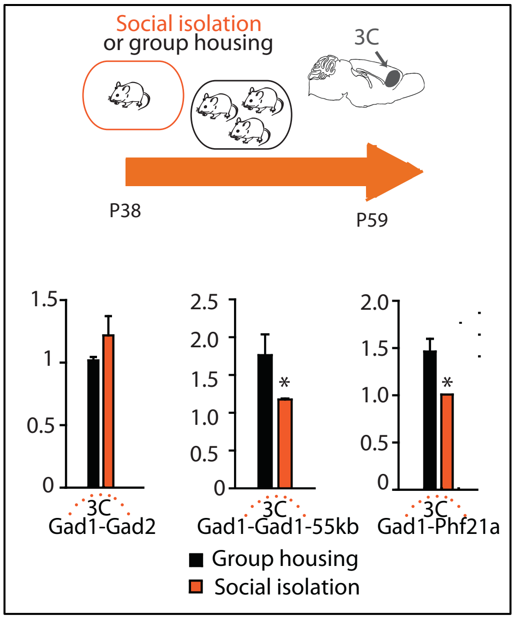

Supplementary Figure 3: Prefrontal 3D genomes after social isolation. (Top) Experimental design and timeline. (Bottom) PFC 3C assays in isolated mice expressed relative to control, N=6 control, N=3 isolated mice. * one tailed t-test, *Gad1-Gad1* 55 kb loop P=0.0414; *Gad1-Phf21a* P=0.021. Data shown as mean±S.E.M.

Supplementary Figure 4

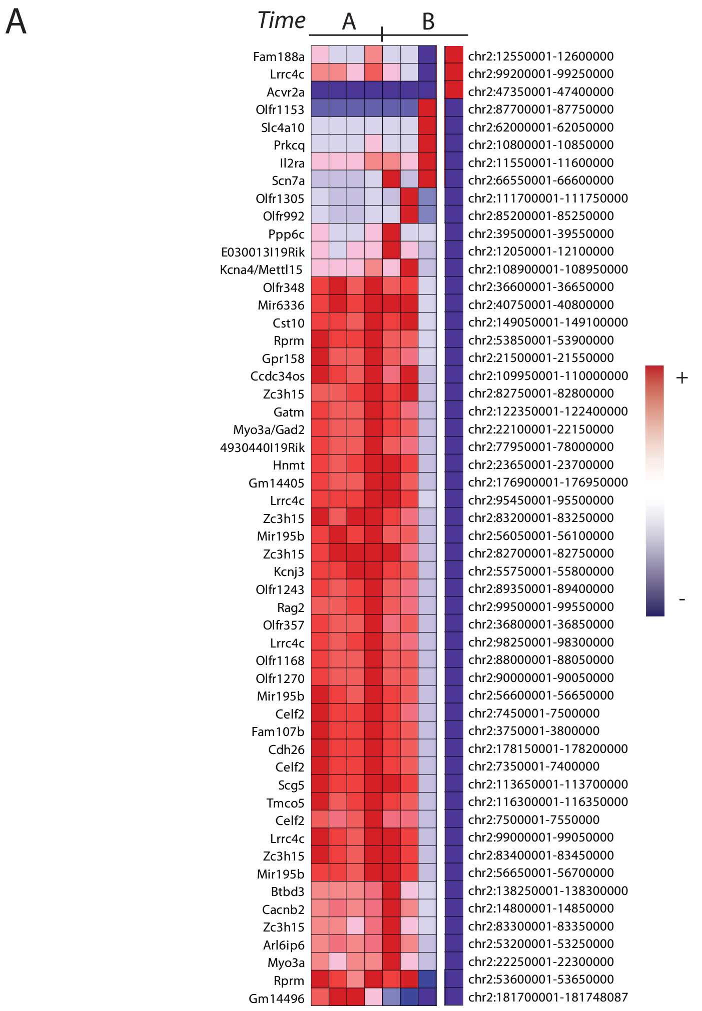

B

		TIME A				TIME B			
		A1	A2	A3	A4	B1	B2	B3	B4
TIME A	A1	1							
	A2	0.988	1						
	A3	0.985	0.999	1					
	A4	0.955	0.900	0.891	1				
TIME B	B1	0.832	0.751	0.736	0.940	1			
	B2	0.706	0.622	0.609	0.823	0.824	1		
	B3	0.112	0.080	0.067	0.177	0.217	0.171	1	
	B4	-0.914	-0.870	-0.859	-0.942	-0.887	-0.795	-0.465	1

Supplementary Figure 4: Correlations among Dam-seq libraries from HSV TALE^{Gad1}DAM-injected PFC. (A) Pearson hierarchical clustering by genomic loci and sample using GenePattern (www.broadinstitute.org) pairwise average-linkage with mean based row and column centering, based on N=4 TIME A and N=4 TIME B libraries. All four TIME A libraries were prepared processed in one batch, and all four TIME B libraries were processed and prepared in a second batch (see Methods for additional information on correlational analyses.Heatmap from +1 (dark red) to -1 (dark blue). (B) Sample-to-sample correlation coefficients, showing robust correlations among 6/8 Dam-seq libraries.

Supplementary Figure 5

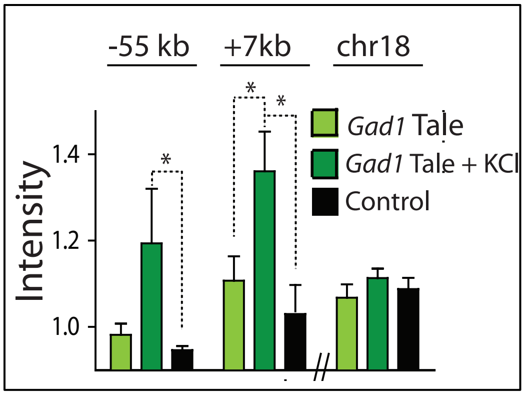

Supplementary Figure 5: Activity-dependent regulation. DamID PCR in primary neuronal culture harvested 72 hours after $TALE^{Gad1}$Dam transfection. Methylated G^{m}ATC tetramers assayed at -55kb and +7kb (from Gad1 TSS), sequence from chromosome 18 as control. N=3-4 neuronal cultures/treatment (KCL or saline administered 6 hours prior to harvest), * P<0.05, two-tailed t-test.

Supplementary Figure 6

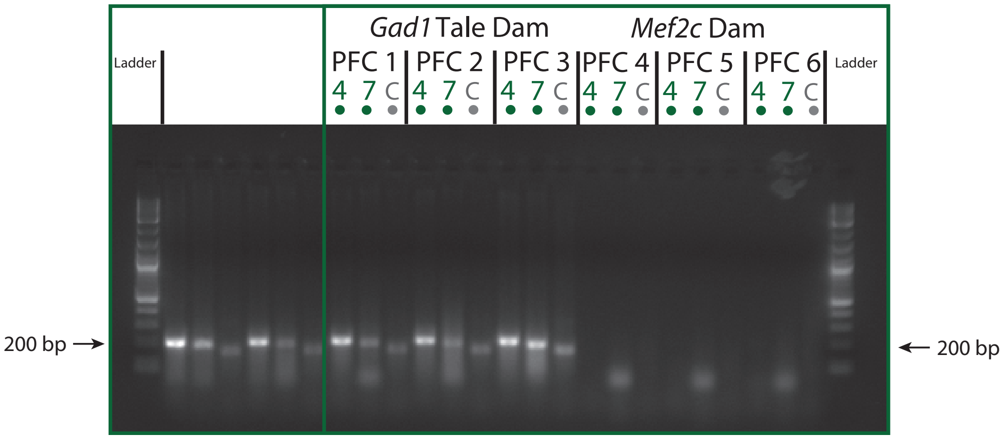

Supplementary Figure 6. **DamID PCR to test sequence-specific G^{m}ATC adenine methylation.** Complete agarose gel picture from loading slots to lane bottom of the gel shown in *Figure 3B* of main manuscript. DamID PCR products are detectable for 55kb *Gad1* loop (primer pair no. 4), corresponding to previously reported loop formation by 3C and for sequences at TALE target sequence (primer pair no. 7) in HSV TALE^{Gad1}Dam injected PFC samples PFC1, PFC2, PFC3. Note absence of DamID PCR product in HSV$^{Mef2c\text{-}Dam}$ injected PFC4, PFC5 and PFC6.

SUPPLEMENTARY TABLE 1. SEQUENCING STATISTICS			
**SAMPLE**	**Adapter (GGTCGCGGCCGAGGATC) Sorted+ Aligned Reads**	**chr2 reads**	**% chr2 reads**
Time A PFC P90 Saline HSV-Gad1-Tale-Dam	1215862	162620	0.212
Time A PFC P90 MK-801 HSV-Gad1-Tale-Dam	673744	120325	0.200
Time A PFC P28 Saline HSV-Gad1-Tale-Dam	686470	263860	0.237
Time A PFC P28 MK-801 HSV-Gad1-Tale-Dam	480690	134662	0.250
Time B PFC P90 Saline HSV-Gad1-Tale-Dam	1072961	103857	0.097
Time B PFC P90 MK-801 HSV-Gad1-Tale-Dam	1008777	111302	0.110
Time B PFC P28 Saline HSV-Gad1-Tale-Dam	399439	103857	0.260
Time B PFC P28 MK-801 HSV-Gad1-Tale-Dam	1620300	168344	0.104
NeuC (Cortical) Gad1-Tale-Dam 1	8865258	460183	0.052
NeuC (Cortical) Gad1-Tale-Dam 2	10422942	794767	0.076
NeuC (Cortical + KCl) Gad1-Tale-Dam 1	16935435	2730853	0.161
NeuC (Cortical + KCl) Gad1-Tale-Dam 2	3830902	367263	0.096
NeuC (Hippocampal) Gad1-Tale-Dam	2772579	4513	0.004
NeuC (Hippocampal) no Gad1-Tale-Dam	216219	555	0.003

SUPPLEMENTARY TABLE 2: Oligonucleotides

Name	Sequence	chr	start	end	Function
Gad1-Tale3	TATTGCCAAGAGAG	chr2	70399332	70399346	TALE
AdRt	CTAATACGACTCACTATAGGGCAGCGTGGTCGCGGCCGAGGA	None in mouse			DamID-seq
AdRb	TCCTCGGCCG				DamID-seq
AdR_PCR	GGTCGCGGCCGAGGATC				DamID-seq
Gad2.mRNAF	TGGAAGCTGAGTGGAGTAGA	Exon spanning			qPCR
Gad2.mRNAR	TCTCTAACCAGGAGAGCTGAA	Exon spanning			qPCR
Gad1.mRNAF	ACATCGACTGCCAATACCAATA	Exon spanning			qPCR
Gad1.mRNAR	CCATCCAACGATCTCTCTCATC	Exon spanning			qPCR
Dam.RNA1F	TCACGCGTACGCGTACCGGCATGAAGAAAAAT				qPCR
Dam.RNA1R	CTATAAGCTTTTATTTTTTCGCGGGTGAAACG				qPCR
Dam.RNA2F	CGCGTACGCGTACCGGCAT				qPCR
Dam.RNA2R	CTATAAGCTTTTATTTTTTCGCGGGTGAAACGACTCC				qPCR
18S.F	TTAGTTGGTGGAGCGATTTG				
18S.R	CACTTGTCCCTCTAAGAAGTTG				
Phf21a.3C	GTCTAACATCATGAATCCAGACCAGCCA	chr2	92008903	92008930	3C
Gad1.3C	GCCTTTGGAAACCAGCGTCCTTCAGTGTTT	chr2	70406539	70406568	3C
Gad2.3C	CCAACACATGTATATGCTAGGTGTGAGGAC	chr2	22456777	22456806	3C
Gad1n.3C	CTGAACCCCAGGGAATAATCGGAT	chr2	70407764	70407787	3C
Gad1-50kb.3C	CTGGACTGGACAGTTGCTATTGCTACCTGA	chr2	70353571	70353600	3C
*	TCTCCTCTTCTGGCTCTGCT	chr18	35989497	35989516	DamID-PCR
*	CTAGTCCGGTCCGGGTTT	chr18			DamID-PCR
4.R / Gad1-50kbR	TAACTGCAGGAAGGCTGAGG	chr2	70353104	70353123	DamID-PCR
4.F / Gad1-50kbF	TCCCTCTGTCTCTCTTCCTCTC	chr2			DamID-PCR
5.R	CCTGTGGTCAGAGCTTTGGT	chr2	70353786	70353805	DamID-PCR
5.F	CCCAATTTGGATAACTGTCACC	chr2	70353608	70353629	DamID-PCR
6.R	GTATCCTAGCCAGTGTTTGTAG	chr2	70364390	70364411	DamID-PCR
6.F	TCAATAAAGAACCTGGGATGG	chr2	70364602	70364622	DamID-PCR
7.R	GGTTTATCCCAGGCCTGTCT	chr2	70398936	70398955	DamID-PCR
7.F	TCGCAAGGAGCAGCTTAGTT	chr2	70398770	70398789	DamID-PCR
8.R	CGGCCTCTTCTAGCCTTCTC	chr2	70399147	70399166	DamID-PCR
8.F	CCCAGAGGTGCACATGAATA	chr2	70398964	70398983	DamID-PCR
Gad1.DamID-PCR.F	CTGAGGTGCGTGGTTTGAG	chr2			DamID-PCR
Gad1.DamID-PCR.R	TCCTTGCGACCAAAGAGAAC	chr2	70398759	70398778	DamID-PCR
Gad1n.DamID-PCR.F	AAACGGGAGTGCAGAAACTC	chr2	70399624	70399643	DamID-PCR
Gad1n.DamID-PCR.R	CCATCCCGTATCTTGGAGAA	chr2	70399803	70399822	DamID-PCR
Phf21a.DamID-PCR.F	GGCCAGATGCTTATTAAGTGGA	chr2	92008443	92008464	DamID-PCR
Phf21a.DamID-PCR.R	GCTCTCCCAAGAGAGGAGTTA	chr2	92008704	92008724	DamID-PCR
Gad2.DamID-PCR.F	AAACACTGCTCCCATTGATAGA	chr2	22444007	22444028	DamID-PCR
Gad2.DamID-PCR.R	CCATTGGCTTCCTAGGCTTTA	chr2	22444312	22444332	DamID-PCR
1.qPCRF	CTTCATGCGAAGCGATCA	chr2	70315121	70315138	DamID-qPCR
1.qPCRR	ATAGGTCCCGCGGATTC	chr2	70315208	70315224	DamID-qPCR
2.qPCRF	CACAGAGCTGGTCTCCT	chr2	70315781	70315797	DamID-qPCR
2.qPCRR	CATCAGATCCCTGGTGAAAC	chr2	70315839	70315858	DamID-qPCR
3.qPCRF	CCTAGAGAGAACAAGAGGGA	chr2	70328656	70328675	DamID-qPCR
3.qPCRR	GACGACTGATGGAAGCTAAG	chr2	70328762	70328781	DamID-qPCR
4.qPCRF	CACTTTCCCTTTGCCTTTATG	chr2	70346224	70346244	DamID-qPCR
4.qPCRR	TTCTGCTATTGCAGGGAAG	chr2	70346294	70346312	DamID-qPCR
5.qPCRF	ACCTTGAATAGTTGAAGAAGCTG	chr2	70361791	70361813	DamID-qPCR
5.qPCRR	GCCTTGGAATTGGTGCTATC	chr2	70361874	70361893	DamID-qPCR
6.qPCRF	AAATATAAGCCTACAGGAGAGC	chr2	70383819	70383840	DamID-qPCR
6.qPCRR	GGTTCTTCTACCATATGGATTCT	chr2	70383890	70383912	DamID-qPCR
7.qPCRF	TGGCATCTTCCACTCCTT	chr2	70401890	70401907	DamID-qPCR
7.qPCRR	CTCGCTGCAGTCCATTTAATA	chr2	70401989	70402009	DamID-qPCR
8.qPCRF	CCTCAGGCTGTATGTCAGA	chr2	70439819	70439837	DamID-qPCR
8.qPCRR	TCAGCTAAGCGAGTCACA	chr2	70439919	70439936	DamID-qPCR
Cacnb2.mRNAF	TAGCTAACCCACCTTATTCAAC	chr2	14846185	14846206	cDNA-qPCR
Cacnb2.mRNAR	GAAGTGGCTGAAGGGATAAG	chr2	14846262	14846281	cDNA-qPCR
My03a.mRNAF	TTAGGCCACCAACTGTAAATAA	chr2	22444108	22444129	cDNA-qPCR
My03a.mRNAR	CTGACTTGCAACCCTACAC	chr2	22444268	22444297	cDNA-qPCR
Phf21a.mRNAF	AACACTACATCTTCATGGGAAA	chr2	92008493	92008514	cDNA-qPCR
Phf21a.mRNAR	TCTCTCCAACGTCCCTTT	chr2	92008610	92008650	cDNA-qPCR
Kcna4.mRNAF	TTAGTTATGGATAATATTGCTGTAGTG	chr2	107546197	107546223	cDNA-qPCR
Kcna4.mRNAR	TCGAGGTTTCTGTCTTATTTGT	chr2	107546317	107546338	cDNA-qPCR
Cxxc5.mRNAF	CCTGAGCTGGGATGTAGA	chr18	35989455	35989472	cDNA-qPCR
Cxxc5.mRNAR	GGTGCGCATTGCTAGG	chr18	35989558	35989573	cDNA-qPCR
